# Validation of the Polish version of the Functional Oral Intake Scale against flexible endoscopic evaluation of swallowing and the International Dysphagia Diet Standardization Initiative Functional Diet Scale

**DOI:** 10.3389/fnut.2025.1524335

**Published:** 2025-02-05

**Authors:** Magdalena Milewska, Barbara Jamroz, Mariusz Panczyk, Joanna Chmielewska-Walczak, Tomasz Czernicki, Marta Dabrowska-Bender, Marcin Folwarski, Dorota Szostak-Wegierek

**Affiliations:** ^1^Department of Clinical Dietetics, Faculty of Health Sciences, Medical University of Warsaw, Warsaw, Poland; ^2^Department of Otolaryngology, National Medical Institute of the Interior and Administration, Warsaw, Poland; ^3^Department of Education and Research in Health Sciences, Faculty of Health Sciences, Medical University of Warsaw, Warsaw, Poland; ^4^Department of Otorhinolaryngology, Head and Neck Surgery, Medical University of Warsaw, Warsaw, Poland; ^5^Department of Neurosurgery and Paediatric Neurosurgery, Medical University of Warsaw, Warsaw, Poland; ^6^Department of Clinical Nutrition and Dietetics, Medical University of Gdańsk, Gdansk, Poland; ^7^Home Enteral and Parenteral Nutrition Unit, General Surgery Department, Nicolaus Copernicus Hospital, Gdansk, Poland

**Keywords:** dysphagia, deglutition, deglutition disorders, functional oral intake, validation, fiberoptic endoscopic evaluation of swallowing

## Abstract

**Introduction:**

The Functional Oral Intake Scale (FOIS) is a widely used instrument for assessing oral intake in dysphagic patients. Despite its frequent use, a validated version for the Polish population has been lacking.

**Methods:**

This study aimed to validate the Polish adaptation of FOIS (FOIS-PL) by examining its concordance with Fiberoptic Endoscopic Evaluation of Swallowing (FEES) outcomes and the International Dysphagia Diet Standardization Initiative Functional Diet Scale (IDDSI-FDS) scores across patients with diverse clinical profiles. The primary outcome measures included the Penetration-Aspiration Scale (PAS) score from FEES, pharyngeal residue quantification, and IDDSI-FDS scores. A total of 302 participants with varying clinical conditions were recruited. The cohort included individuals with head and neck malignancies, cerebrovascular incidents, neuromuscular disorders, and other dysphagia aetiologies.

**Results:**

Patients with gastroesophageal reflux disease and those post-thyroidectomy consistently exhibited oral food intake with a FOIS-PL score of ≥5. A strong inverse correlation was found between FOIS-PL scores and PAS scores (rho = −0.739; *p* < 0.001), indicating that reduced oral intake was associated with increased penetration or aspiration risk. Significant differences in FOIS-PL scores were evident across patient subgroups stratified by PAS severity (PAS ≤ 2, PAS 3–5, PAS > 5) and IDDSI levels. Lower FOIS-PL scores corresponded with more impaired swallowing safety (PAS > 5). The median FOISPL score was 5 for individuals with pharyngeal residue and 6 for those without (*p* < 0.001). Inter-rater reliability between evaluations conducted by a dietitian (FOIS I) and a speech-language pathologist (FOIS II) demonstrated high consistency (tau = 0.995; *p* < 0.001). Convergent validity was supported by strong correlations between FOIS-PL and IDDSI-FDS scores (FOIS I vs. IDDSI-FDS I: tau = 0.819; *p* < 0.001; FOIS II vs. IDDSI-FDS II: tau = 0.815; *p* < 0.001).

**Conclusion:**

The Polish version of the Functional Oral Intake Scale (FOIS-PL) is a valid and reliable tool for assessing oral intake in dysphagia. The findings demonstrate high accuracy, reliability, and validity, supporting its use across diverse clinical conditions.

## Introduction

Dysphagia refers to difficulty in swallowing saliva, fluids, solid foods, or drugs, and may involve any of the four stages of deglutition: the oral preparatory (or processing), oral (transport), pharyngeal, and esophageal phases. The process of swallowing (deglutition) involves cortical centers of both cerebral hemispheres, the swallowing center located in the brainstem, cranial nerves (V, VII, IX, X, and XII), and sensory receptors located at the level of the pharynx. The oral phase involves both oral processing, i.e., chewing food and mixing it with saliva to form a bolus, and the subsequent propelling of the bolus towards the back of the tongue (the oral transport phase). The moment the base of the tongue touches the back of the throat is when the involuntary, pharyngeal phase starts. The pharyngeal phase involves the raising of the hyoid bone, retroflexion of the epiglottis (to protect the airways), the closing of the vocal folds, contraction of pharyngeal walls (to propel the bolus), and relaxation of the upper esophageal sphincter. Any disruption of this process may result in dysphagia (aspiration, silent aspiration, or penetration) or ineffective swallowing (e.g., drooling or saliva retention in the valleculae and/or piriform sinuses) (1–3). These types of dysfunctions are evaluated with methods considered to be the gold standard of dysphagia diagnostics, namely videofluoroscopic swallow study (VFSS) and fiberoptic endoscopic evaluation of swallowing (FEES) (4, 5). Dysphagia may affect, e.g., patients with tumors of the head and neck, those with a history of an ischemic stroke, traumatic brain injury, surgery of the neck and chest, neuromuscular disorders, or neurodegenerative conditions. Primary consequences of dysphagia include malnutrition, dehydration, and aspiration pneumonia, whereas secondary consequences are hospital readmissions, institutionalization, and loss of functional status (2). Management of dysphagia may include rehabilitation, the use of maneuvers that increase the safety of swallowing, and compensatory techniques that involve modifying the consistency of liquid and solid foods or administering food via an alternative route (via a nasogastric tube or gastrostomy) (6). Since instrumental swallowing assessment methods are still largely unavailable in clinical practice, validated questionnaires can be used to quickly obtain an initial assessment of the severity of dysphagia and select a safe ingestion method (7). The Functional Oral Intake Scale (FOIS) was originally validated for a group of patients with neurogenic dysphagia, but it is currently used in various patient populations. The FOIS is a tool that helps quickly assess the problem to determine the safe route of feeding. Scores from 1 to 3 indicate tube feeding, whereas scores from 4 to 7 indicate total oral diet, with or without special preparation. This scale is a functional rating scale and helps assess changes over time due to disease progression or therapeutic interventions (8).

The FOIS has been validated against the instrumental methods VFSS and FEES only in its Chinese (9), German (10), Italian (11), and Persian (12) language versions. The remaining validations reflected cross-cultural adaptation (13) or construct validity (14) and validity based on overall results or against questionnaires similar to the FOIS (15). The purpose of our study was to validate the Polish version of FOIS both against FEES and the International Dysphagia Diet Standardisation Initiative Functional Diet Scale (IDDSI-FDS) scores in patients with heterogeneous clinical conditions to optimize clinical management of dysphagia.

## Materials and methods

This study was divided into two steps. The first step was the translation process, and the second step was dedicated to the study of the validity and reliability of the Polish version of the Functional Oral Intake Scale (FOIS-PL).

### Translation

Permission for translation was obtained from the author of the original FOIS version (8).

The translation process was conducted according to the WHO guidelines (16). The established translation committee consisted of members representing different specializations. Each expert was an active clinician and researcher in the field of dysphagia. Moreover, advanced linguistic and cultural skills were required.

Translation of the original FOIS into Polish was performed by a dietitian, a phoniatrician, and a speech and language pathologist (SLP), who deal with dysphagia diagnosis and treatment and have proficient competencies in written and spoken English. Parallel translations were conducted. A review of the first proposals of translation was randomly assigned to the translators and two reviewers to indicate discrepancies and identify any misunderstandings between the original and translated versions. A final version of FOIS-PL was proposed. Next, back translation was performed by a translator, who did not know FOIS. Any further differences were discussed by the expert committee to approve the final version of FOIS-PL. The obtained complete version was pretested on 15 consecutive patients admitted to the Swallowing Lab. No doubts or discrepancies between the SLP’s and the dietitian’s assessments were observed (Table 1).

**Table 1:** The original functional oral intake scale (FOIS) and a polish translation of the FOIS (FOIS-PL).

FOIS level	FOIS (original version)	Poziom FOIS-PL	FOIS-PL (translation)
Level 1	Nothing by mouth	Poziom 1	Nic doustnie
Level 2	Tube dependent with minimal attempts of food or liquid	Poziom 2	Żywienie dojelitowe z niewielkimi ilościami pokarmu lub płynu doustnie
Level 3	Tube dependent with consistent oral intake food or liquid	Poziom 3	Żywienie dojelitowe z uzupełniającym żywieniem drogą doustną (pokarmy lub płyny)
Level 4	Total oral diet of a single consistency	Poziom 4	Pełne żywienie doustne jedną konsystencją
Level 5	Total oral diet with multiple consistencies, but requiring special preparation or compensation	Poziom 5	Pełne żywienie doustne kilkoma konsystencjami, ale specjalne przygotowanie lub metody kompensacyjne są wymagane
Level 6	Total oral diet with multiple consistencies, without special preparation but with specific food limitations	Poziom 6	Pełne żywienie doustne kilkoma konsystencjami bez specjalnego przygotowania, ale pewne produkty są wykluczone
Level 7	Total oral diet with no restrictions	Poziom 7	Pełne żywienie doustne bez ograniczeń

### Validation process

This study was carried out according to the Declaration of Helsinki and was approved by the Ethics Committee of the Medical University of Warsaw (no. AKBE/224/2018). Ethical approvals and participant consent were obtained following international and national guidelines. Demographic, clinical, and instrumental data were gathered prospectively for an ongoing study on swallowing disorders in different clinical settings.

The validation process was based on analyzing the dietary history and FEES score of consecutive patients admitted to the Swallowing Disorders Laboratory of a teaching hospital (Warsaw, Poland) in the period 2018–2022. The study inclusion criteria were dysphagia or suspected dysphagia, age of >18 years, normal cognitive function, and written informed consent. Patients were divided into 8 groups: Group 1. – Patients diagnosed with head and neck cancer; Group 2 – patients after stroke; Group 3 – patients diagnosed with neuromuscular diseases; Group 4 – post-traumatic brain injury patients; Group 5 – patients suffered from gastroesophageal reflux diseases (GERD); Group 6 – patients after thyroidectomy; 7 – patients diagnosed with head and neck paraganglioma; Group 8 – other dysphagia etiologies (e.g., post-intubation, rare genetic diseases, sarcopenic dysphagia).

For our validation study, we used the following outcome measures: the penetration–aspiration scale (PAS) score for FEES, a pharyngeal residue, and IDDSI-FDS. The study was conducted by experts with over 6-year experience in dysphagia.

### Inter-rater reliability

Before performing FEES, we took each patient’s dietary history, focusing on the amount and consistency of ingested fluids and solids as well as the feeding route (oral, enteral). Subsequently, the speech-language pathologist (SLP) and the dietitian were asked to assign the FOIS-PL scores to all patients based on the collected dietary records. To reduce the effect of dietary changes over time on FOIS scores, all analyses were conducted on the day of the FEES procedure. Raters were unable to consult one another about the findings. Since high inter-rater agreement was likely, kappa Cohen’s coefficient was used.

### Cross-validation

Cross-validation was conducted in 302 patients based on the presence of symptoms indicating compromised swallowing safety (based on the PAS) and impaired swallowing efficiency (based on pharyngeal residue) found on FEES. All FEES assessments were conducted by a single phoniatrician with instructor qualifications. The PAS is an 8-point scale, with a score of 1 representing no penetration or aspiration, 2 indicating transient penetration with ejection; 3–5 representing laryngeal penetration without ejection and/or reaching the vocal folds, and scores of 6–8 representing aspiration. For this study PAS scores of 1–2 were considered normal, PAS scores of >2 were considered as penetration and/or aspiration, with PAS scores of 3–5 showing penetration (17), and PAS scores of >5 showing aspiration (18). Swallowing efficiency was measured solely via a dichotomous scale (pharyngeal residue—yes/no). The lowest PAS score for each of the evaluated food consistencies and pharyngeal residue (irrespective of consistency) was considered in our analysis.

### Convergent – validation

In addition, two raters (a dietitian and SLP) evaluated IDDSI-FDS scores in light of FEES findings. The IDDSI-FDS rates the severity of dysphagia depending on the extent of consistency modification both in liquid and solid foods. The greater the consistency-related limitations the lower the IDDSI-FDS score. Moreover, the resulting score, which reflects the relationship between the recommended consistency for solids and that for liquids shows the number of possible levels of food consistency that can be administered to the patient (19).

### FEES methodology

The phoniatrician (BJ) performing the FEES test is an experienced specialist and holder of a FEES Instructor Certificate from the European Society of Swallowing Disorders (ESSD). The FEES examination was performed based on a Polish protocol designed in the Medical University of Warsaw Otolaryngology Clinic based on ESSD and IDDSI guidelines, literature analysis, and authors’ personal experience. The protocol starts with a clinical assessment of swallowing (a history of swallowing problems, a screening test, and qualification of the patient for FEES). The procedure was divided into 3 stages:

Stage 1. Anatomy and physiology of the nose, nasopharynx, oropharynx, hypopharynx, and larynx.

Stage 2. Assessment of swallowing saliva and food and liquids of different consistencies according to the IDDSI classification.

Stage 3. Evaluation of the effectiveness of therapeutic maneuvers (20).

To assess swallowing safety, we used the PAS (18). PAS scores of 1–2 were considered normal. If a score of 7 or higher was achieved, the assessment was discontinued.

The FEES examination was performed with Xion nasofiberoscope (XION GmbH, Berlin, Germany), 3,2 mm diameter, light source, camera, and color monitor following a unified study protocol (20). The protocol of administering individual consistencies depended on the patient’s general condition, reported symptoms, and any problems observed during the examination itself, e.g., compromised swallowing safety or markedly impaired swallowing efficiency. The swallowing of saliva and the ability to control fluid in the oral cavity were assessed in each patient. Fluids of various IDDSI-specified consistencies were prepared with a Nutilis Clear xanthan gum thickener, according to the manufacturer’s instructions. Typically, the examination was initiated by administering a mildly thick liquid (IDDSI level 2) in incremental volumes of 5, 10, and 20 mL and, depending on the determined swallowing safety, other liquid consistencies were assessed (thin and slightly thick, IDDSI levels 0 and 1, respectively, or extremely thick, IDDSI level 4). Subsequently, minced and moist foods (IDDSI level 5, e.g., oatmeal combined with thick applesauce, lump size 4 mm), soft and bite-sized foods (IDDSI level 6, e.g., a 1.5 × 1.5 cm banana bite), and regular, easy to chew foods (IDDSI level 7), e.g., a crackers, were evaluated. The protocol was modified whenever swallowing safety was impaired for a specific consistency and volume or significantly impaired at an “easier” consistency.

Each FEES was assessed by the same phoniatrician and SLP, and decisions about the route of feeding and the possibility of achieving efficient hydration and nutrition orally were additionally consulted with a dietitian.

It is worth noting that decisions regarding the feeding route and oral food consistency depended not only on the raw FEES score, but also on the level of patient self-sufficiency, the possibility of preparing food of the required consistency, the likelihood of patient adherence to recommendations on adequate hydration and nutrition.

### Statistical analysis

The methodology of this study incorporated both descriptive and inferential statistics to rigorously validate the FOIS-PL and elucidate the relationships and differences within the data. Descriptive statistics provided a comprehensive overview of the demographic and clinical characteristics of the 302 participants. These statistics also depicted the distribution of individual FOIS-PL scores, using histograms to visually represent the percentage of patients at each score level, highlighting variations among groups with different conditions.

Non-parametric tests, specifically the Mann–Whitney U and Kruskal-Wallis tests, were employed to compare FOIS-PL scores across different subgroups defined by their PAS scores. These tests are well-suited for analyzing ordinal data or data not meeting normal distribution assumptions, effectively assessing differences in median FOIS-PL scores across various levels of swallowing impairment.

Additionally, Spearman’s rank correlation coefficient was used to explore the association between FOIS-PL and PAS scores.

The study also focused on the interrater reliability and convergent validity of the FOIS-PL, evaluating the consistency of assessments conducted by different healthcare professionals. Kendall’s tau coefficient was applied to determine the concordance between these ratings.

Throughout the analyses, a significance level of 0.05 was maintained, ensuring that findings deemed statistically significant had a less than 5% probability of occurring by chance. The statistical analyses were executed using Jamovi (Version 2.5; Computer Software, Sydney, Australia).

## Results

### Patient characteristics

A total of 302 participants were included in the present study. The detailed demographic and clinical characteristics of the study sample are presented in Table 2.

**Table 2:** Demographic and clinical characteristics of the study group (*N*; %).

	Total		FOIS 1		FOIS 2		FOIS 3		FOIS 4		FOIS 5		FOIS 6		FOIS 7	
	*N*	%	*N*	%	*N*	%	*N*	%	*N*	%	*N*	%	*N*	%	*N*	%
Gender																
F	168	55.6	15	8.9	13	7.7	7	4.2	3	1.8	68	40.5	39	23.2	23	13.7
M	134	44.4	17	12.7	22	16.4	8	6.0	1	0.7	39	29.1	25	18.7	22	16.4
Group																
1	120	39.7	16	50.0	16	45.7	11	73.3	2	50.0	35	32.7	25	39.1	15	33.3
2	26	8.6	6	18.8	3	8.6	1	6.7	0	0.0	10	9.3	3	4.7	3	6.7
3	32	10.6	2	6.3	4	11.4	0	0.0	0	0.0	15	14.0	8	12.5	3	6.7
4	14	4.6	2	6.3	3	8.6	0	0.0	0	0.0	6	5.6	3	4.7	0	0.0
5	15	5.0	0	0.0	0	0.0	0	0.0	0	0.0	2	1.9	4	6.3	9	20.0
6	18	6.0	0	0.0	0	0.0	0	0.0	0	0.0	6	5.6	6	9.4	6	13.3
7	40	13.2	2	6.3	3	8.6	0	0.0	1	25.0	21	19.6	9	14.1	4	8.9
8	37	12.3	4	12.5	6	17.1	3	20.0	1	25.0	12	11.2	6	9.4	5	11.1

Group 1 – head and neck cancer; Group 2 – stroke; Group 3 – neuromuscular diseases; Group 4 – brain injury; Group 5 – GERD; Group 6 – thyroidectomy; 7 – paraganglioma; Group 8 – other dysphagia etiologies. F-female; M – male; N – number of cases.

**Figure 1:**
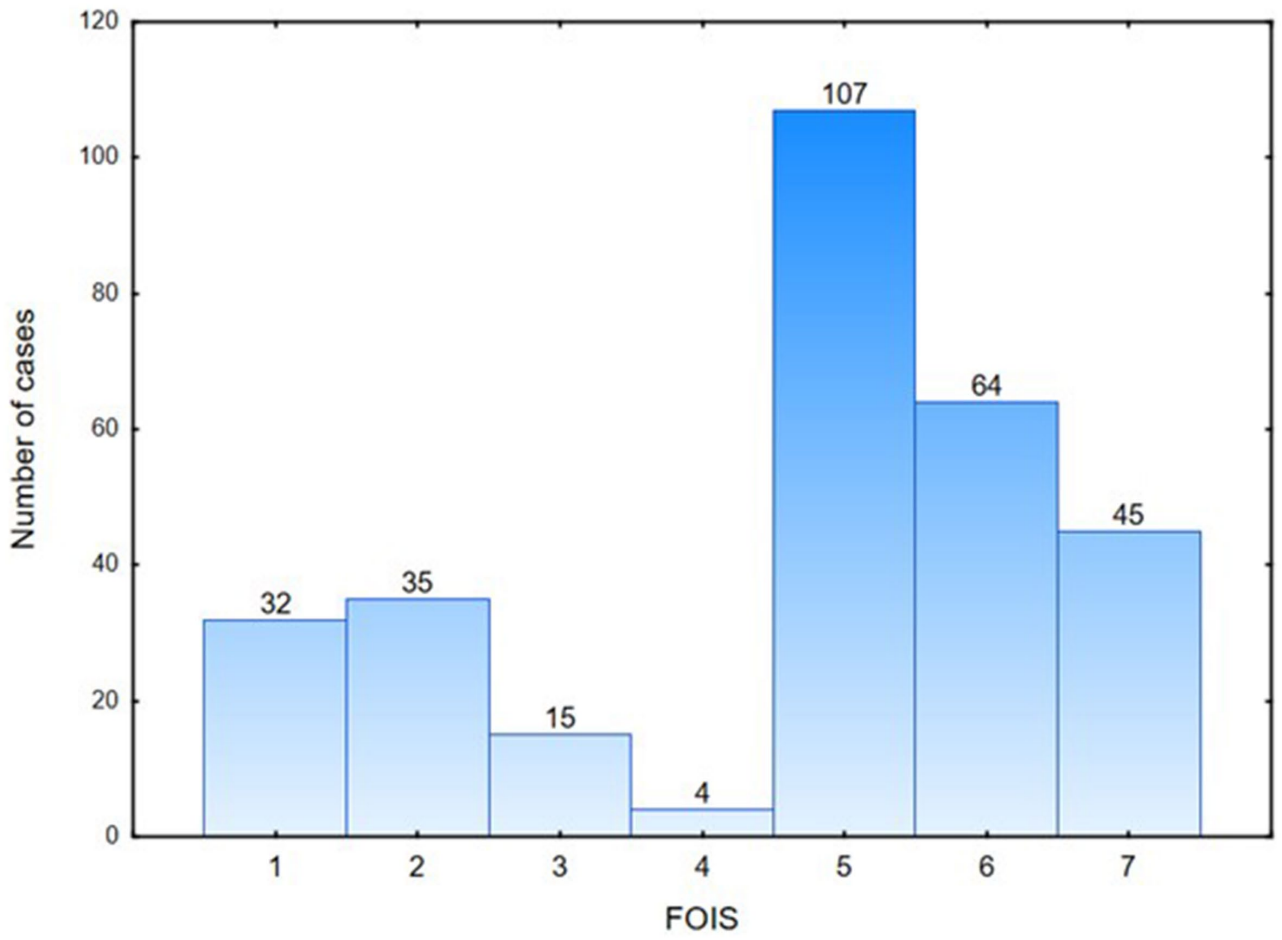
General functional oral intake scale -PL results distribution (*N* = 302).

**Figure 2:**
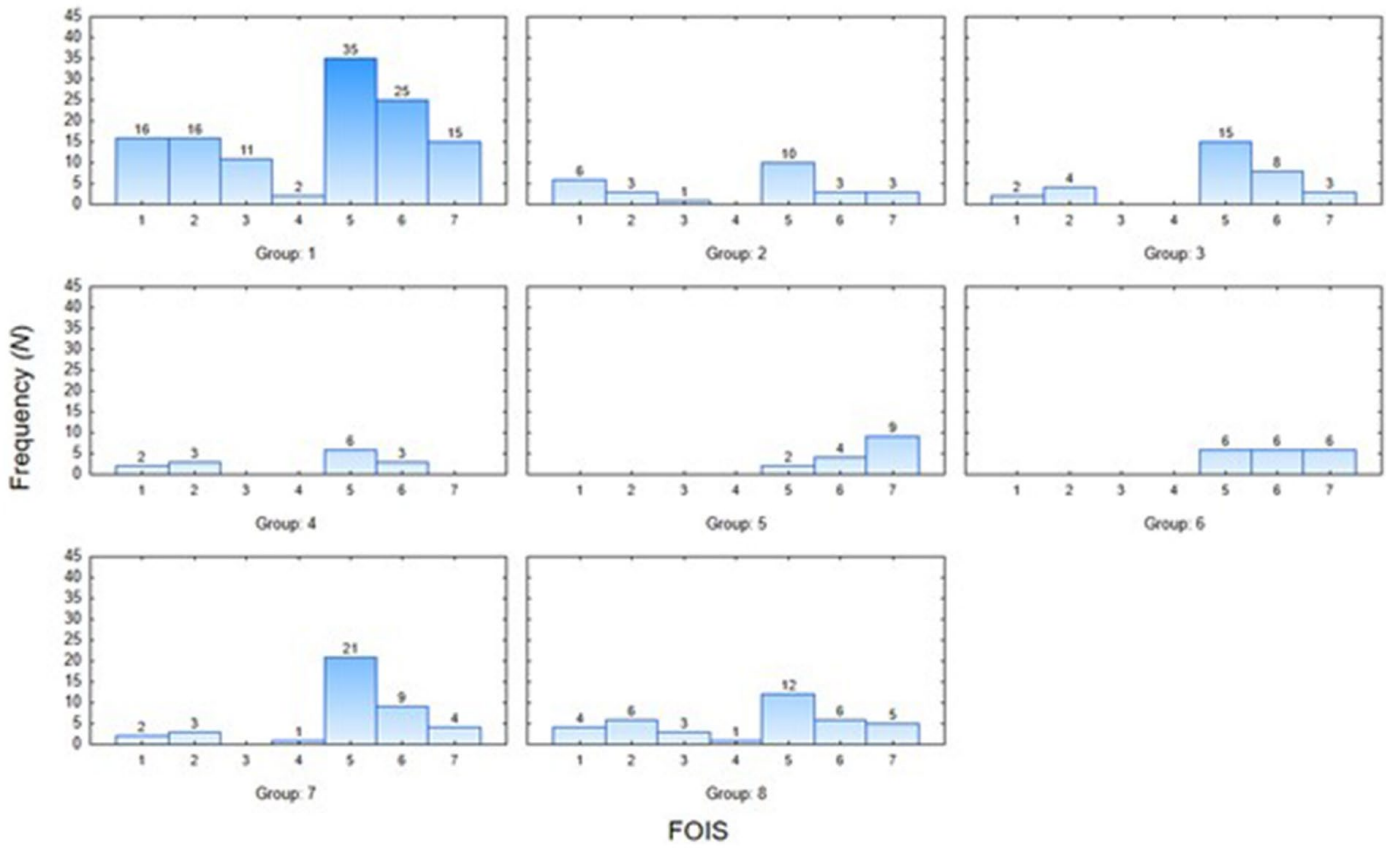
Functional oral intake scale-PL results distribution in subgroups (*N* =302).

Figure 1 shows the percentage distribution of the individual FOIS-PL scores for the entire study population, with 27% of patients requiring tube feeding, and 14.9% of patients exhibiting safe and effective oral feeding, with no consistency limitations (FOIS-PL 7) (Figure 1). The widest variety in FOIS-PL scores was observed in the subgroup of patients with tumors of the head and neck, a history of stroke, other dysphagia causes, and neuromuscular conditions, whereas all patients with gastroesophageal reflux disease and those following thyroidectomy had oral food intake (FOIS-PL ≥ 5). The majority of patients with tumors of the head and neck, neuromuscular conditions, stroke, head trauma, or paraganglioma achieved a FOIS-PL score of 5. Patients diagnosed with gastroesophageal reflux disease typically showed no restrictions as to their food intake (FOIS-PL score of 7) (Figure 2).

### Cross-validation

#### FOIS and swallowing safety

Penetration or aspiration (PAS scores >2) was most often observed for IDDSI consistency levels of 0 (*n* = 153; 50.66%) and 1 (*n* = 121; 40%). The rates of penetration and aspiration observed with individual consistencies are presented in Table 3. The variety in the numbers of patients evaluated with individual consistencies is due to the study design and patient pre-assessment for their ability to safely ingest foods of the so-called “difficult” consistencies (IDDSI levels 0, 1, 6, and 7), and their ability to ingest anything orally. The study protocol, including the choice of tested consistencies, was adopted for each patient and depended on the results of the Clinical Swallowing Evaluation and the first stage of FEES protocol. There was a strong negative correlation (rho = −0.739; *p* < 0.001) between FOIS-PL and PAS worst score. Analysis of individual consistencies revealed a strong negative correlation between FOIS and PAS for IDDSI levels 0 and 1, whereas a moderate correlation for all other IDDSI levels (3–7) (Table 4). Statistically significant differences in the distribution of FOIS-PL scores were observed between every subgroup of patients stratified by PAS scores (PAS ≤ 2; PAS 3–5; PAS > 5), at all IDDSI levels, with a clear tendency towards a lower median FOIS-PL score in the group with severely impaired swallowing safety (PAS > 5). The median FOIS-PL score in that last group was 2 for IDDSI consistency levels of 0, 2, and 3, and 1 for consistency levels 5, 6, and 7.

**Table 3:** Results of the Mann– Whitney U and Kruskal–Wallis tests for the comparison of FOIS-PL between patients with and without penetration/aspiration (PAS > 2), penetration (PAS > 2 ≤ 5), and aspiration (PAS > 5) on FEES.

Bolus type based on IDDSI	Sign of dysphagia	FOIS_PL			
		N	Mdn (IQR)	MW test/KW test	*p*-value
Level 0	Penetration/aspiration				
	PAS ≤ 2	121	6.0 (6.0–7.0)	10.582	<0.001
	PAS > 2	153	5.0 (2.0–6.0)		
	Penetration				
	PAS ≤ 2	121	6.0 (6.0–7.0)	140.793	<0.001
	PAS > 2 ≤ 5	76	5.0 (5.0–6.0)		
	PAS > 5	77	2.0 (1.0–5.0)		
	Aspiration				
	PAS ≤ 5	197	6.0 (5.0–7.0)	10.344	<0.001
	PAS > 5	77	2.0 (1.0–5.0)		
Level 1	Penetration/aspiration				
	PAS ≤ 2	143	6.0 (6.0–7.0)	10.875	<0.001
	PAS > 2	121	5.0 (3.0–5.0)		
	Penetration				
	PAS ≤ 2	143	6.0 (6.0–7.0)	134.033	<0.001
	PAS > 2 ≤ 5	60	5.0 (5.0–6.0)		
	PAS > 5	61	3.0 (2.0–5.0)		
	Aspiration				
	PAS ≤ 5	203	6.0 (5.0–7.0)	9.667	<0.001
	PAS > 5	61	3.0 (2.0–5.0)		
Level 2	Penetration/aspiration				
	PAS ≤ 2	218	6.0 (5.0–7.0)	10.268	<0.001
	PAS > 2	61	2.0 (2.0–4.0)		
	Penetration				
	PAS ≤ 2	218	6.0 (5.0–7.0)	112.787	<0.001
	PAS > 2 ≤ 5	27	4.0 (2.0–5.0)		
	PAS > 5	34	2.0 (1.0–2.0)		
	Aspiration				
	PAS ≤ 5	245	6.0 (5.0–7.0)	9.154	<0.001
	PAS > 5	34	2.0 (1.0–2.0)		
Level 3	Penetration/aspiration				
	PAS ≤ 2	215	6.0 (5.0–7.0)	8.742	<0.001
	PAS > 2	43	2.0 (2.0–4.0)		
	Penetration				
	PAS ≤ 2	215	6.0 (5.0–7.0)	83.403	<0.001
	PAS > 2 ≤ 5	20	4.5 (2.5–5.5)		
	PAS > 5	23	2.0 (1.0–2.0)		
	Aspiration				
	PAS ≤ 5	235	6.0 (5.0–7.0)	8.000	<0.001
	PAS > 5	23	2.0 (1.0–2.0)		
Level 4	Penetration/aspiration				
	PAS ≤ 2	209	6.0 (5.0–7.0)	8.583	<0.001
	PAS > 2	45	2.0 (2.0–5.0)		
	Penetration				
	PAS ≤ 2	209	6.0 (5.0–7.0)	81.257	<0.001
	PAS > 2 ≤ 5	25	5.0 (3.0–5.0)		
	PAS > 5	20	1.5 (1.0–2.0)		
	Aspiration				
	PAS ≤ 5	234	6.0 (5.0–7.0)	7.544	<0.001
	PAS > 5	20	1.5 (1.0–2.0)		
Level 5	Penetration/aspiration				
	PAS ≤ 2	195	6.0 (5.0–7.0)	6.073	<0.001
	PAS > 2	24	5.0 (2.0–5.0)		
	Penetration				
	PAS ≤ 2	195	6.0 (5.0–7.0)	39.726	<0.001
	PAS > 2 ≤ 5	18	5.0 (4.0–5.0)		
	PAS > 5	6	1.0 (1.0–1.0)		
	Aspiration				
	PAS ≤ 5	213	6.0 (5.0–7.0)	4.379	<0.001
	PAS > 5	6	1.0 (1.0–1.0)		
Level 6	Penetration/aspiration				
	PAS ≤ 2	174	6.0 (6.0–7.0)	5.973	<0.001
	PAS > 2	32	5.0 (4.5–6.0)		
	Penetration				
	PAS ≤ 2	174	6.0 (6.0–7.0)	38.757	<0.001
	PAS > 2 ≤ 5	28	5.0 (5.0–6.0)		
	PAS > 5	4	1.0 (1.0–1.0)		
	Aspiration				
	PAS ≤ 5	202	6.0 (5.0–7.0)	3.608	<0.001
	PAS > 5	4	1.0 (1.0–1.0)		
Level 7	Penetration/aspiration				
	PAS ≤ 2	159	6.0 (6.0–7.0)	6.271	<0.001
	PAS > 2	37	5.0 (5.0–6.0)		
	Penetration				
	PAS ≤ 2	159	6.0 (6.0–7.0)	42.502	<0.001
	PAS > 2 ≤ 5	31	5.0 (5.0–6.0)		
	PAS > 5	6	1.0 (1.0–5.0)		
	Aspiration				
	PAS ≤ 5	190	6.0 (5.0–7.0)	3.959	<0.001
	PAS > 5	6	1.0 (1.0–5.0)		

MW test—Mann– Whitney U test; KW test—Kruskal–Wallis test; Mdn – median; IQR – interquartile range; PAS – Penetration-Aspiration Scale.

**Table 4:** Spearman correlation between FOIS and PAS for each IDDSI level.

Pair of variables	Spearman rank correlation coefficient			
	N	rho Spearman	*t*	*p*-value
FOIS_PL & PAS_Level 0	274	−0.72	−17.197	<0.001
FOIS_PL & PAS_Level 1	264	−0.74	−17.765	<0.001
FOIS_PL & PAS_Level 2	279	−0.66	−14.535	<0.001
FOIS_PL & PAS_Level 3	258	−0.56	−10.868	<0.001
FOIS_PL & PAS_Level 4	254	−0.57	−10.932	<0.001
FOIS_PL & PAS_Level 5	219	−0.42	−6.910	<0.001
FOIS_PL & PAS_Level 6	206	−0.42	−6.667	<0.001
FOIS_PL & PAS_Level 7	196	−0.45	−7.063	<0.001

FOIS_PL – polish version of functional oral intake scale; PAS – penetration-aspiration scale; *t*-value from the *t*-test evaluating the significance of the Spearman correlation coefficient.

#### FOIS and swallowing efficiency

Analysis of swallowing efficiency found 148 patients (49.3%) to have a residue (irrespective of its consistency) in the valleculae or piriform sinuses. The median FOIS-PL was 5 in the individuals with a residue and 6 in those without (*p* < 0.001). According to the adopted Polish study protocol, the residue-related information was only about the presence, or absence, of a residue regardless of its exact location or the tested consistency. Therefore, we were unable to consider the various IDDSI consistencies in comparing the FOIS-PL scores in patients with a residue with those without.

#### Interrater reliability and convergent validity of IDDSI-FDS test scores

Interrater reliability was assessed based on the degree to which the FOIS-PL scores assigned by 2 independent investigators (an SLP and a dietitian) were consistent. This assessment showed very high rates of consistency in FOIS I (conducted by dietician) and FOIS II (conducted by SLP) (tau 0.995; *p* < 0.001). Due to the heterogeneity of the study group in terms of the stage of treatment at the time of study inclusion, we decided that estimating test–retest reliability might be burdened with a high bias during the second measurement.

The remaining coefficients of concordance between the two measurements (FOIS and IDDSI-FDS) conducted by independent investigators indicated excellent interrater reliability (Table 5) We observed a high degree of consistency between IDDSI-FDS I (performed by a dietician) and IDDSI-FDS II (performed by SLP) measurements (tau 0.999; *p* < 0.001). Convergent validity was assessed based on the relationship between FOIS-PL and IDDSI-FDS test scores (FOIS I vs. IDDSI-FDS I tau 0.819; *p* = 0.0000 and FOIS II vs. IDDSI-FDS II tau 0.815; *p* = 0.0000).

**Table 5:** Coefficients of concordance between the two measurements.

Kappa Cohena	Pi Scotta	Kappa Fleissa	Alfa Krippendorfa
Coefficients of concordance between the two measurements FOIS—PL I and FOIS- PL II			
0.97	0.97	0.96	1.00
Coefficients of concordance between the two measurements IDDSI-FDS I and IDDSI-FDS II			
0.99	0.99	0.98	1.00

FOIS-PL I – Polish version of Functional Oral Intake Scale performed by the dietician; FOIS-PL II– Polish version of Functional Oral Intake Scale performed by the speech-language pathologist; IDDSI-FDS I—IDDSI-FDS assessment performed by dietician; IDDSI-FDS II—IDDSI-FDS assessment performed by the speech-language pathologist.

**Figure 3:**
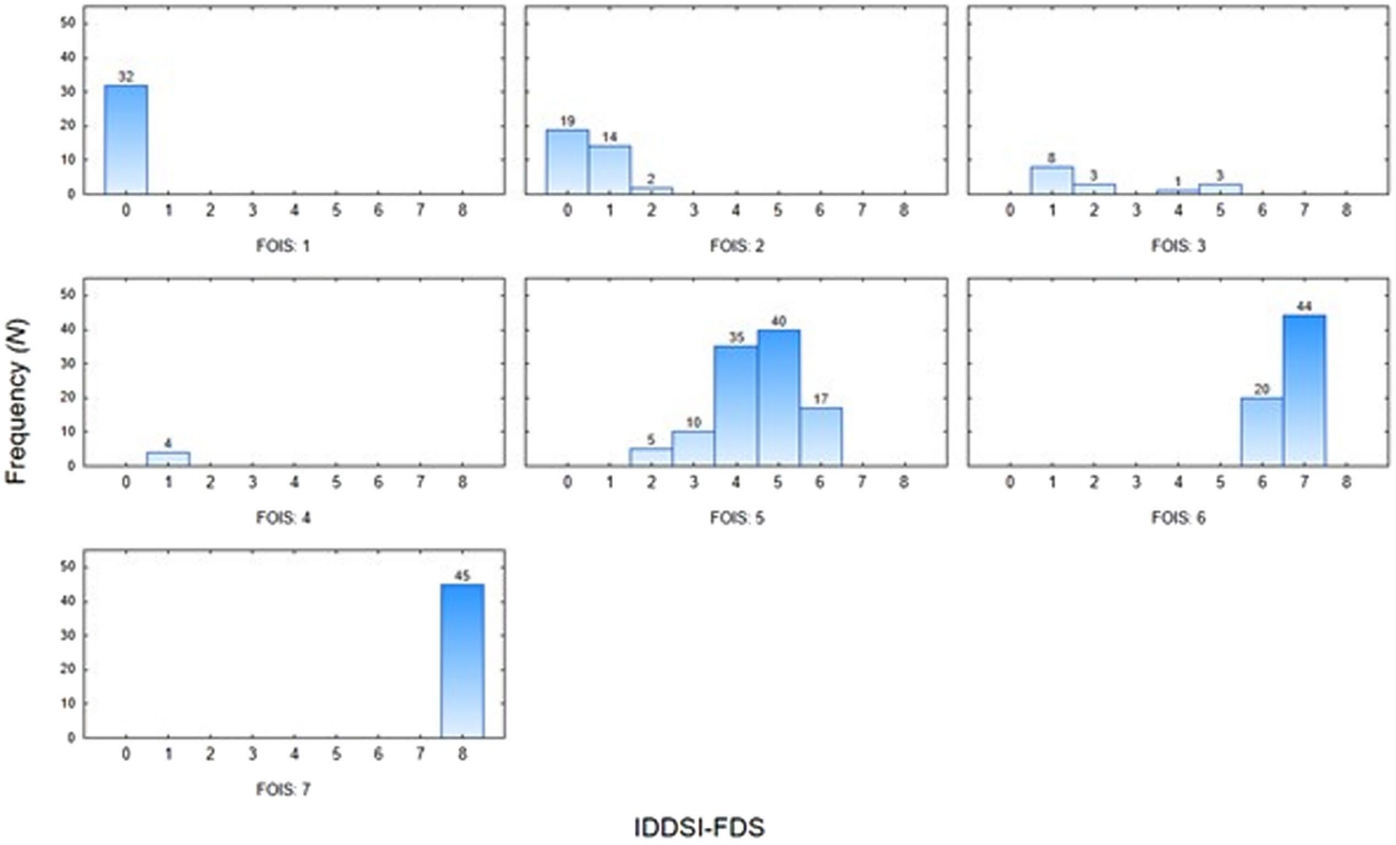
The pairings of FOIS-PL and IDDSI FDS scores.

Moreover, we analyzed the most common pairings of FOIS-PL and IDDSI-FDS scores. Patients who scored 1 or 2 in the FOIS-PL were most likely to score 0 in the IDDSI-FDS, whereas those who scored 3 or 4 in the FOIS-PL were most likely to score 1 in the IDDSI-FDS. In patients with a FOIS-PL score of 5, the predominant IDDSI-FDS score was 5. A FOIS-PL score of 6 was most commonly associated with an IDDSI FDS score of 7, and a FOIS-PL score of 7 was only observed in patients with an IDDSI FDS score of 8 (Figure 3).

## Discussion

The FOIS is the most commonly used tool for functional assessment of deglutition both in research studies and in clinical practice. Although the FOIS was originally designed for stroke patients, we decided to expand the study group to include patients with acute and chronic course of diseases. Therefore, the purpose of our study was to validate the Polish FOIS version against FEES (cross-validation) and IDDSI-FDS (convergent validity) in a population with dysphagia of various etiology. According to the consensus-based standards for the selection of health measurement instruments (COSMIN) (21) construct validity can be evaluated based on convergent validity and known-group validity. We did not analyze interrater reliability due to study population heterogeneity and the impossibility of conducting measurements at the same intervals (the patients were treated in various centers throughout Poland and were not always available for measurements). For the same reasons, we did not analyze sensitivity to change. Nonetheless, several authors agreed that the FOIS (in both the original language version and translations to various languages) is sensitive to changes in the feeding method and route over time (8, 9, 12). To the best of our knowledge, our study is the third one, after an Italian (11) and a German (10) study, that used the instrumental FEES method to assess swallowing safety and effectiveness. We also chose the IDDSI-FDS based on the fact that our study protocol used IDDSI-specified food consistencies. The introduction of a Polish-language version of this tool will help standardize assessment results in various centers, not only those within Poland but also those abroad. This approach will facilitate creating consistent standards of treatment. The FOIS-PL was translated following international guidelines and validated by experienced investigators. The fact that all FOIS-PL scores were represented in such a heterogeneous study population indicates the usefulness of this tool in other populations, which is consistent with reports by other authors (10, 11). The patients included in our study were consecutive patients who were scheduled to undergo a FEES, and were at various stages of treatment for etiologically diverse conditions, which made the study group so heterogeneous. We believe another study is needed, one where the study group would be stratified into pre- and postoperative patients and the stage of the advancing of condition would be considered. Moreover, the FEES conducted as part of our study was based on a unique protocol, which may additionally affect the results of the assessments and make it difficult to compare our results with those of other authors. To our knowledge, ours was the third FOIS-PL validation study using IDDSI-specified consistencies. Considering the widespread use of the IDDSI system worldwide, there is a growing need to adapt the existing tools to the new classification of food and liquid consistencies. We would also like to emphasize the fact that this was the first validation study that included a dietitian as part of the team of investigators making decisions on the treatment and prevention of malnutrition and dehydration. Obtaining a complete dietary history, including the method of intake and the risk of dehydration and malnutrition, helps rationally determine FOIS levels. We believe that, apart from the patient’s clinical status and intake method, this determination should be based on the patient’s nutritional status, ability to meet energy and protein requirements through oral feeding, ability to achieve adequate hydration, and willingness to cooperate, including the degree of independence. Mortensen et al. also suggested that the decision regarding enteral nutrition had been most commonly based on difficulties in maintaining wakefulness, impaired control of the torso and head, and malnutrition. Those authors concluded that the FOIS is a good tool for assessing dysphagia; however, the selection of an alternative (non-oral) feeding route should be based on other factors that can affect feeding effectiveness and safety. The authors of the original FOIS version also pointed out that, while determining FOIS levels, it is essential to consider to what extent the patient’s nutritional needs are met with oral feeding; the authors suggested analyzing 3-day dietary diaries or obtaining history from family members or guardians (8). The European Society for Clinical Nutrition and Metabolism (ESPEN) guidelines regarding malnourished patients or those at a high risk of malnutrition may help in deciding on introducing enteral feeding. Nutrition via other routes (rather than oral) is indicated when there is no possibility of oral feeding for 7 days or oral food intake is expected to cover less than 60% of estimated needs for 7–14 days. Additional criteria are a loss of body weight of >5% over 1–3 months, a catabolic state, or a period of chemotherapy (22). In our daily practice, we follow the guidelines established based on a FEES study whose protocol included IDDSI-specified liquid and solid food consistencies and enteral nutrition guidelines in combination with assessing the risk of malnutrition and dehydration. To avoid discrepancies and facilitate discussing our results with other authors, in this study we classified liquids as IDDSI levels 0, 1, 2, 3, and 4, semisolids as IDDSI level 5, and solids as IDDSI levels 6 and 7 (23, 24). FOIS level 4 was applied in a few ratings, which is consistent with that reported by Mortensen et al. (14). Most of our patients required enteral nutrition despite partial oral nutrition, and by the time when the decision to remove the tube was made, the patients were typically able to ingest food of more than one consistency; this is why we omitted level 4. In light of the widespread use of IDDSI-specified consistencies, it seems worthwhile to adapt the existing tools to the new classification of liquid and solid food consistencies.

In line with our suppositions, the FOIS-PL proved to be a highly accurate and reliable tool, which is consistent with reports by other authors (9–12). Notably, the large sample size used in our study augments the robustness of our findings. We validated FOIS against the PAS scale obtained in FEES. Previous studies to validate the FOIS against instrumental methods (FEES or VFSS) used English-, Chinese-, German-, Italian-, and Persian-language versions of the scale (8–12). FEES tests are much more commonly used in Poland than VFSS, which increases the clinical significance of our study. We showed a strong negative correlation between the compromised swallowing safety assessed via the PAS scale and FOIS. Hamzic et al. (10) and Zhou et al. (9) reported similar findings, whereas Ninfa et al. (11) demonstrated a moderate correlation. These discrepancies are likely to be due to the heterogeneity of the compared groups and the differences in study methodologies. The similarity between the results of cross-validation against VFSS or FEES reported in the literature and those found in our study supports the high accuracy of both methods. Those study groups that exhibited aspiration in the case of all the evaluated IDDSI-specified consistencies had median FOIS-PL scores of 1–3, which indicates total or partial enteral nutrition. In a study by Ninfa et al. (11) the median values for groups of patients exhibiting aspiration with all evaluated consistencies were higher and ranged from 4 to 5. In our study, the decisions to introduce enteral nutrition were made more commonly in patients whose dietary history suggested a high risk of aspiration or in those who had been referred to undergo the assessment when they were on enteral nutrition. Patients who exhibited a residue showed a median FOIS-PL of 5, irrespective of the evaluated food consistency, which is consistent with observations by Ninfa et al. (11). Moreover, those authors reported a significant lowering of FOIS scores in patients with a massive pyriform sinuses residue. Unfortunately, the FEES test protocol we used did not call for identifying residue location or amount, which certainly is a limitation of our study. Importantly, the measured FOIS level was based exclusively on observing the patient deal with various consistencies during the examination without introducing any maneuvers to enhance swallowing safety or reduce residue, with only the lowest level considered in further analysis. In summary, patients showing both compromised safety and impaired effectiveness of swallowing were assigned lower FOIS levels.

Our study used the IDDSI-specified consistencies and, therefore, convergent validation was performed with IDDSI- FDS. To our knowledge, only Vogrinčič et al. (15) used the IDDSI framework and the IDDSI-FDS scale in their studies. We would like to emphasize that differences in study designs between different countries are due to the lack of a standardized approach to both diagnostics and treatment, which was also pointed out by Prikkladnicki et al. (25). Despite the fact, that we used the increasingly more commonly used IDDSI classification in our study, our study protocol included no scales that took into account residue location or amount, which limited the comprehensiveness of our assessment of swallowing efficiency.

Inter-rater reliability assessments were conducted by two different specialists, in a blinded way. As far as we know, this is the first validation study of the FOIS with a dietitian among the investigators, which helped collect a complete dietary history. We believe that the inclusion of other healthcare professionals into research teams expands the diagnostic potential, and taking a thorough dietary history facilitates reliable specialist assessments. We decided to test convergent validity with the IDDSI-FDS, a tool that is recommended when using IDDSI-specified food and liquid consistencies. We demonstrated a strong positive correlation between the scales (FOIS and IDDSI FDS), which is consistent with findings by Vogrinčič et al. (15). Moreover, analysis of our results helped us identify the most common pairings of IDDSI-FDS and FOIS-PL scores. Patients with higher FOIS-PL scores were shown to score higher in the IDDSI-FDS, which indicated the possibility of using a greater number of different consistencies of liquid and solid food. A validation study by the original creators of the IDDSI-FDS also demonstrated a strong positive correlation with FOIS levels (19). A similar Slovenian study evaluated a ten times smaller study population, making it difficult to discuss the results. Nonetheless, case-by-case data analysis showed that FOIS level 5, for example, was most associated with an IDDSI-FDS score of 5 (despite the IDDSI-FDS scores ranging from 3 to 7). We obtained the same observations however, IDDSI-FDS scores ranged from 2 to 6. The authors of the original IDDSI-FDS showed that a FOIS score of 5 most often corresponded to IDDSI-FDS scores of 5–6 (19). These discrepancies may be due to either substantial differences in study group size and characteristics or differences in the decision-making process as to which IDDSI-specified consistencies are safe after instrumental tests had been conducted.

### Strengths of the study

To our knowledge, this study was the first attempt at validating FOIS based both on an instrumental method (FEES) and the IDDSI-FDS. The inclusion of a dietitian into the team of investigators was likely to more comprehensively assess each patient when determining their FOIS level. Since we intended to use the best clinical practices, our decision-making as to the route of feeding and the amount of solid and liquid foods was based not only on the safety of swallowing but also on the prevention of malnutrition and dehydration, and the willingness of the patient to cooperate, including their degree of independence. This approach was reflected in the resulting IDDSI-FDS scores. Like the authors of an Italian study (11), we believe it advisable to include the body mass index, weight loss over time, the nature of the underlying condition, and the achieved proportion of the estimated nutritional requirements into FOIS-based analyses. These factors may significantly affect functional oral intake.

## Limitations

Our study has some limitations. Undoubtedly, one limitation of our study was the fact that neither intra-rater reliability nor sensitivity to change could be evaluated. Conducting a longitudinal study with a more homogenous patient cohort or including repeated assessments by a single rater over time could be implemented in future research. These approaches would provide deeper insights into the scale’s responsiveness and intra-rater consistency. Another potential, limitation was the fact that our study protocol differed from the protocol of the original study when the original FOIS was being validated. Moreover, FOIS-PL validation was conducted at a single center, which may have increased the degree of agreement between the investigators. Therefore, we recommend that a multicenter study should be designed. Nonetheless, our study was conducted at a center specializing in dysphagia treatment in collaboration with a multidisciplinary team. The fact that patients with dysphagia of various etiologies were included also suggests that the determination of FOIS levels was based on other factors besides swallowing safety and effectiveness. It is advisable to conduct analyses in subgroups stratified by the underlying conditions. Moreover, due to the diverse study protocols, including the various classifications of liquid and solid food consistencies, it is difficult to compare findings. Perhaps, the use of the FOIS-PL and IDDSI-FDS by the same investigators may have also led to the high degree of their agreement.

## Conclusion

The FOIS is an assessment tool characterized by high accuracy and reliability. This tool has been used both in clinical practice and research studies. The Polish version of the FOIS questionnaire closes a current diagnostic gap in Poland. However, we recommend further studies to address the limitations of our study mentioned above. Due to the widespread use of IDDSI-specified consistencies, it seems advisable to adapt the existing tools to the IDDSI classification of liquid and solid food consistencies.

## Data Availability

The raw data supporting the conclusions of this article will be made available by the authors, without undue reservation.
